# Recurrent fibroepithelial vaginal polyp in a 2-year-old girl: a case report and review of the literature

**DOI:** 10.1097/MS9.0000000000001836

**Published:** 2024-02-15

**Authors:** Mohammad Hakam Shehadeh, Ahmad M. Abualrub, Waleed M. Malhes, Amar Msarweh, Wael Amro

**Affiliations:** a Faculty of Medicine, Al-Quds University, Jerusalem; b Department of Pediatric Surgery, Palestine Medical Complex (PMC), Ramallah, Palestine

**Keywords:** child, fibroepithelial polyp, recurrent, vagina

## Abstract

**Introduction and importance:**

Fibroepithelial vaginal polyps (FEPV) are rare mucosal polypoid lesions primarily found in adult women. However, FEPV in paediatric patients, especially beyond the neonatal period, is exceedingly uncommon. Documenting cases improves diagnosis and management. FEPV can mimic malignancy, posing challenges for interpretation. Early detection, treatment, and follow-up are crucial for optimal outcomes.

**Case presentation:**

The authors present the case of a 2-year-old girl with a recurrent FEPV. The patient had a painless, reddish, smooth, soft, rubbery polypoid vaginal mass measuring 2.3 × 1.5 cm. Physical examination revealed no concurrent systemic issues. Surgical excision of the polyps confirmed them as fibroepithelial polyps. Despite previous excisions, the polyps recurred within a month. The patient’s postoperative recovery was uneventful, and subsequent follow-up showed no recurrence.

**Clinical discussion:**

FEPV presents as mucosal polypoid lesions with a connective tissue core covered by benign squamous epithelium. It is rare before menarche and after menopause. Although the pathophysiology remains unclear, hormonal factors and local injuries may contribute. FEPV is usually asymptomatic but may cause pressure, obstruction, bleeding, or discharge. Differential diagnosis includes vaginal connective tissue malignancies. The diagnosis was confirmed by surgical excision and histopathology. Complete excision is crucial for preventing recurrence.

**Conclusion:**

This case report highlights recurrent FEPV in a 2-year-old girl. Despite previous excisions, polyps recurred, emphasizing the need for complete excision. Documenting cases will enhance our understanding. Further research is needed to elucidate the pathogenesis of paediatric FEPV. Early detection, treatment, and follow-up are essential for optimal management.

## Introduction

Highlights

- Fibroepithelial vaginal polyps (FEPV) is characterized by mucosal polypoid lesions with a connective tissue core covered by a benign squamous epithelium. It is rarely seen before menarche and after menopause, emphasizing the significance of documenting such cases for improved diagnosis and management. The pathophysiology remains unclear, but hormonal factors and local injury to the vaginal mucosa are believed to contribute to its development.
- FEPV can mimic the clinical presentation of malignancy, leading to challenges in interpretation. Early detection, appropriate treatment, and long-term follow-up are crucial to ensure optimal outcomes.
- The presented case involves a two-year-old girl with recurrent FEPV. Despite previous excisions, the polyps recurred, highlighting the need for complete excision to prevent recurrence.
- Clinically, FEPV is usually asymptomatic but can cause symptoms such as pressure, obstruction, bleeding, or discharge. Differential diagnosis includes malignant tumours originating from the vaginal connective tissue.
- Surgical excision is performed to exclude malignancy, and histopathology confirms the diagnosis. Recurrence following incomplete excision has been reported, highlighting the importance of complete excision.
- Documentation of rare cases, such as recurrent FEPV in paediatric patients, contributes to the understanding of the condition. Further research is required to elucidate the pathogenesis of FEPV in paediatric patients.
- Early detection, appropriate treatment, and long-term follow-up are essential for optimal management of FEPV.

Fibroepithelial vaginal polyps (FEPV) in newborns or infant girls are exceedingly rare^1^. Although it is benign, it can sometimes mimic the clinical presentation of sarcoma botryoides, and its histologic features may exhibit atypical characteristics that resemble malignancy, further complicating the clinical interpretation.

This article presents a case report involving a rare case of a two-year-old girl with recurrent vaginal polyps. The purpose of this report is to highlight the clinical presentation, diagnostic evaluation, and management of this particular case, underscoring the significance of early detection, appropriate treatment, and long-term follow-up in similar cases. This case report has been reported in line with the SCARE Criteria^2^.

### Case presentation

A 2-year-old girl was presented to our hospital with a recurrent vaginal polypoid mass that was initially discovered by her parents 8 months ago, the parents reported that the vaginal mass was intermittently disappearing. The patient did not experience any abnormal vaginal discharge or bleeding and had no impact on bowel or urine function. Furthermore, the patient did not present with any concurrent systemic issues. She was delivered at 40 weeks of gestation through routine spontaneous vaginal delivery, and the pregnancy proceeded without complications.

The patient was considered the second child of a healthy father and mother who suffered from polycystic ovary syndrome (PCOS), aged 38 and 33 years, respectively, and there was no family history of malignancies or genitourinary anomalies.

**Figure 1:**
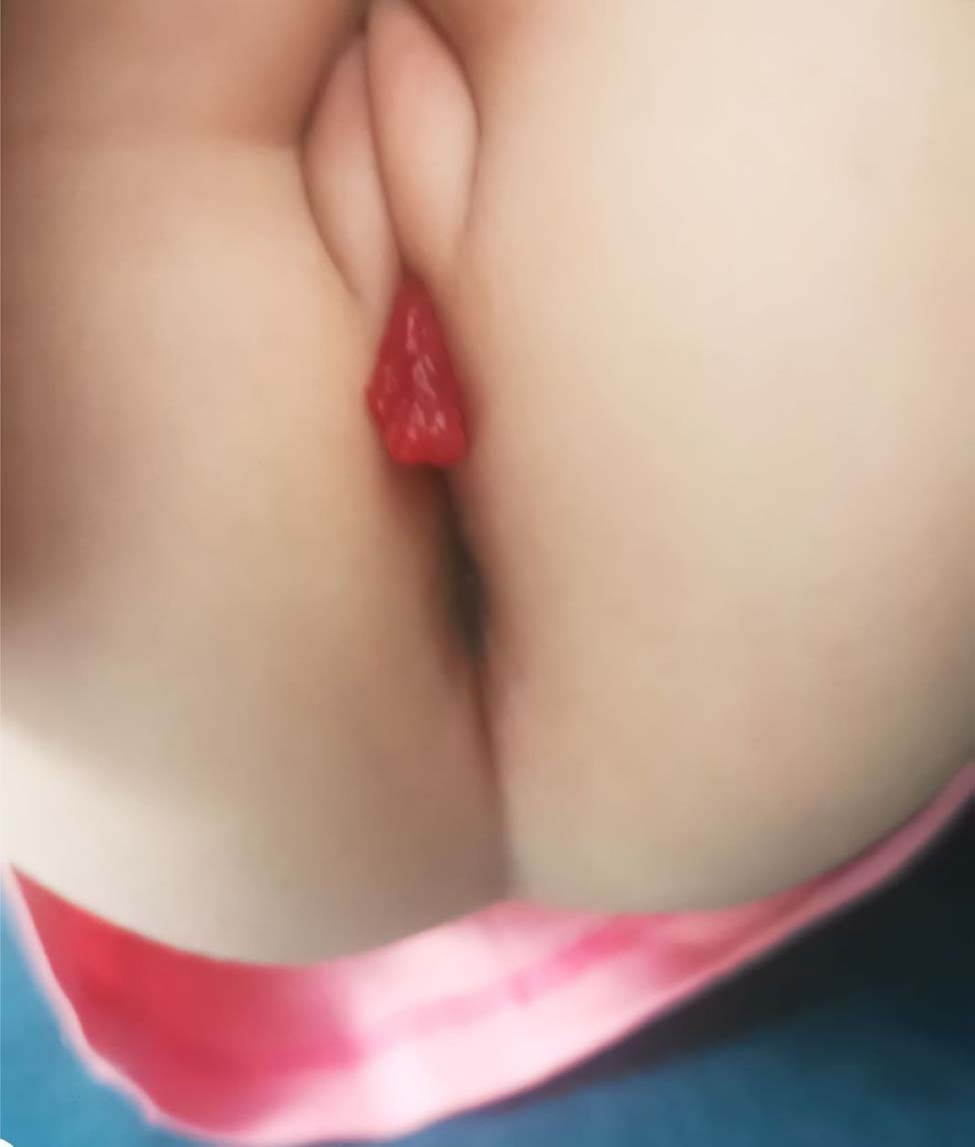
A 2.3 ×1.5 cm polypoid mass, originating from the vaginal wall.

Physical examination revealed a painless, reddish, smooth, soft to rubbery polypoid vaginal mass measuring 2.3 × 1.5 cm. The mass originated from the midline of the posterior vaginal wall [Figure 1], and the urethral and anal openings appeared normal. Additional examination findings were unremarkable.

Ultrasound and MRI established that the circumscribed ovoid homogeneous mass originated from the distal vagina, measuring 2.3 × 1.5 cm mass without any invasion to the adjacent organs.

Prior to the current presentation, the patient underwent two surgical excisions at a different hospital. The first surgical excision took place one week after the discovery of the mass, while the second excision occurred three months after the initial excision. However, each excision was followed by polyp recurrence after one month.

Cystoscopy and vaginoscopy were performed by a paediatric urologist together with a paediatric surgeon under general anaesthesia, which revealed a pedunculated vaginal mass originating from the midline point of the posterior vaginal wall. Apart from the presence of the mass, the cystoscopy and vaginoscopy findings were normal. The mass was completely surgically excised with a margin of 3 mm around its base, emphasizing the protection of the hymenal ring and the patient recovered uneventfully after the procedure.

Histopathological examination confirmed a fibroepithelial polyp lined by a benign squamous epithelium with an underlying stroma. The stroma showed hypercellular and hypocellular areas containing spindled to ovoid neoplastic cells surrounding the dilated thin-walled vessels (highlighted by CD31 and ERG-1). Immunohistochemically, the neoplastic cells were positive for CD34 and STAT-6 and focally positive for ER and smooth muscle actin (SMA). They were negative for PAN-CK, EMA, P53 (wildtype/non-mutant), Desmin, Myogenin, Myo-D1, SALL-4, inhibin, CD99, BCOR, PAN-TRK, CD31, protein S100, and SOX10. INI-1/SMARCB1 and BRG1/SMARCA4 were retained.

Vaginoscopy was performed after 3 months of follow-up, and there was no evidence of recurrence.

## Discussion

FEPV are mucosal polypoid lesions with a connective tissue core covered by a benign squamous epithelium^3^. In 1966, Norris and Taylor first documented the fibroepithelial polyp (FEP) and theorized that it represents a manifestation of maternal or placental hormonal influence in newborn infants, analogous to clitoral or breast enlargement and thickening of the vaginal mucosa^4^. Although FEPV primarily occurs in adult women, it is occasionally observed during pregnancy. However, it is exceedingly rare before menarche and after menopause^5^. The pathophysiology of FEPV remains elusive; however, it is believed to result from a granulation tissue reaction following some local injury of the vaginal mucosa ^6,7^.

Clinically, these polyps are usually asymptomatic and commonly detected during postnatal medical examinations or noticed by the family. However, they can cause symptoms such as feeling of pressure, obstruction of the vagina or urethra, vaginal bleeding, and discharge^8^. Despite their benign nature, they may display a bizarre histological appearance, raising concerns about the possibility of misdiagnosis as malignant tumours originating from the vaginal connective tissue. The differential diagnosis for a vaginal mass in a prepubertal girl includes sarcoma botryoides, rhabdomyosarcoma, and mixed mesodermal tumours^9^. Surgery is primarily performed to exclude the presence of malignancy, and histopathology confirms the diagnosis. FEPV polyps have not been reported to undergo malignant transformation or metastasis.

FEVP should be considered for interlabial masses in prepubertal girls^1^. Treatment is simple local excision, and recurrence is extremely rare^10^. Nevertheless, there have been reported cases of recurrence following incomplete excision^5^, which we suspect may have occurred in our case.

The rarity of fibroepithelial polyps and their recurrence in paediatric patients emphasize the importance of documenting such cases for improved diagnosis and management. The literature review revealed seven cases of FEPV in newborn girls. Although these reported cases were infants, our case is the third case of FEPV in a paediatric patient beyond the neonatal period. A comparison between these cases and the current case is presented in [Table 1]. Due to the limited number of reported cases of FEPV in paediatric patients, further research is needed to shed light on their pathogenetic mechanisms.

**Table 1:** Comparison of cases reported in literature with current case

Author	Case presentation	Management	Follow-up
Norris and Taylor^4^	^a^Infant with grapelike masses protruding from introitus at birth ^b^Infant with soft nodule 0.5 cm in diameter just inside the hymenal ring	^a^Biopsy ^b^Local excision	^a^Masses regressed: no recurrence 8 years ^b^No recurrence 8 months
PUL *et al*.^1^	Newborn with vaginal polyp	Pathology after excision revealed Fibrovascular stroma and acanthotic multilayered squamous epithelium	No recurrence
Jallouli *et al.* ^12^	Newborn with vaginal polyp	Surgical excision was done, and histology revealed FEPV	Follow-up for 10 months reveals no recurrence
Alotay *et al*.^13^	Neonate with interlabial mass	Histology revealed FEPV. treated by surgical excision with 3 mm margins	1 year of follow-up reveals no recurrence
Suzen *et al*.^14^	2-year-old female with vaginal polyp	Surgical excision was done, and histology revealed FEPV	1 year of follow-up reveals no recurrence
Thomas *et al*.^15^	Newborn with vaginal polyp	Surgical excision was done, and histology revealed FEPV	No recurrence
Smart *et al*.^16^	Newborn with 2 polypoid vaginal masses	Complete resection was done. Pathology revealed FEPV	No recurrence
Boreikaitė *et al*.^17^	8-year-old female with papillomatosis and FEPV	Histology revealed FEPV and negative examination for HPV	No recurrence
Current case	2-year-old female with vaginal polyp	Complete resection was done. Pathology revealed FEPV	1 month of follow-up reveals recurrence (×2) 3 months of follow-up reveals no recurrence

FEPV, fibroepithelial polyp of the vagina; HPV, human papillomavirus.

a First case.

b Second case.

It is worth mentioning that their presence, especially recurrence, can cause distressing symptoms for the patient and his family and raise concerns. A suspected diagnosis of genital organ cancer in a woman’s medical history is associated with an elevated risk of developing depression, anxiety, and adjustment disorders^11^.

## Conclusion

Future research on these lesions is needed to shed light on their pathogenetic mechanisms, particularly in paediatric patients. Additionally, healthcare providers should be aware of the possibility of FEPV in young children. Complete surgical excision is recommended for appropriate management. However, incomplete resection may result in disease recurrence, which emphasizes the importance of long-term follow-up.

## Ethical approval

Our institution has exempted this study from ethical review.

## Consent

Written informed consent was obtained from the patient's parents/legal guardian for publication and any accompanying images. A copy of the written consent is available for review by the Editor-in-Chief of this journal on request.

## Sources of funding

Not applicable.

## Author contribution

Writing the manuscript: M.H.S., A.A., W.M., A.M. Reviewing and editing the manuscript: W.A.

## Conflicts of interest disclosure

All authors have no conflict of interest to declare.

## Research registration unique identifying number (UIN)

Not applicable.

## Guarantor

Wael Amro.

## Data availability statement

Not applicable.

## Provenance and peer review

Not commissioned, externally peer-review.

## References

[1] Pul M Yilmaz N Gürses N. Vaginal polyp in a newborn—a case report and review of the literature. Clin Pediatr (Phila) 1990;29:346. 2361345 10.1177/000992289002900612

[2] Sohrabi C Mathew G Maria N. The SCARE 2023 guideline: updating consensus Surgical CAse REport (SCARE) guidelines. Int J Surg Lond Engl 2023;109:1136. 10.1097/JS9.0000000000000373 PMC10389401 37013953

[3] Halvorsen TB Johannesen E. Fibroepithelial Polyps of the Vagina: Are They Old Granulation Tissue Polyps? J Clin Pathol 1992;45:235–240. 1556233 10.1136/jcp.45.3.235 PMC495483

[4] Norris HJ Taylor HB. Polyps of the vagina. A benign lesion resembling sarcoma botryoides. Cancer 1966;19:227–232. 5905466 10.1002/1097-0142(196602)19:2<227::aid-cncr2820190214>3.0.co;2-w

[5] Sharma S Albertazzi P Richmond I. Vaginal polyps and hormones—is there a link? A case series. Maturitas 2006;53:351–355. 16029937 10.1016/j.maturitas.2005.06.007

[6] Tanos V Berry KE Seikkula J. The management of polyps in female reproductive organs. Int J Surg 2017;43:7–16. 28483662 10.1016/j.ijsu.2017.05.012

[7] Song JS Song DE Kim KR. Cellular pseudosarcomatous fibroepithelial stromal polyp of the vagina during pregnancy: a lesion that is overdiagnosed as a malignant tumor. Korean J Pathol 2012;46:494–498. 23136578 10.4132/KoreanJPathol.2012.46.5.494 PMC3490121

[8] Ramírez Melgar E Kunhardt Urquiza E Romero Arauz J. [Fibroepithelial polyp of the vagina. Report of a case]. Ginecol Obstet Mex 2000;68:368–370. 11080942

[9] Putran J Gupta R. Vaginal polyp: an unusual cause of postmenopausal bleeding. Gynecol Surg 2011;8:49–50.

[10] Burt RL Prichard RW Kim BS. Fibroepithelial polyp of the vagina. A report of five cases. Obstetr Gynecol 1976;47:52S–54S. 1246395

[11] Kostev K Jacob L Kalder M. Risk of depression, anxiety, and adjustment disorders in women with a suspected but unconfirmed diagnosis of breast or genital organ cancer in Germany. Cancer Causes Control 2017;28:1021–1026. 28856543 10.1007/s10552-017-0948-1

[12] Jallouli M Trigui L Gargouri A. Vaginal polyp in a newborn. Eur J Pediatr 2008;167:599–600. 17619202 10.1007/s00431-007-0524-x

[13] Alotay AA Sarhan O Alghanbar M. Fibroepithelial vaginal polyp in a newborn. Urol Ann 2015;7:277–278. 25835034 10.4103/0974-7796.152952 PMC4374277

[14] Erturk N Celik OI Suzen A. Fibroepithelial polyp of vagina in a two-year-old girl and review of the literature. Pediatr Urol Case Rep 2016;3:52–52. doi:10.14534/pucr.2016216038

[15] Thomas P Rai P. Vaginal fibroepithelial polyp in a neonate: a rare case. Eur J Obstet Gynecol Reprod Biol 2016;203:340. 10.1016/j.ejogrb.2016.05.052 27365215

[16] Smart VA Roger EI Ricca RL. Fibroepithelial vaginal polyps in a newborn female. Urology 2019;132:161–163. 31255540 10.1016/j.urology.2019.06.026

[17] Boreikaitė E Bilius V Bumbul-Mazurek E. Squamous vaginal papillomatosis in prepubertal female twins: a case report. Acta Med Litu 2021;28:374–378. 35474933 10.15388/Amed.2021.28.2.18 PMC8958654

